# Views Most Would Never See

**DOI:** 10.3201/eid3009.AC3009

**Published:** 2024-09

**Authors:** Byron Breedlove

**Keywords:** Winslow Homer, On the Trail, about the cover, art–science connection

**Figure:**
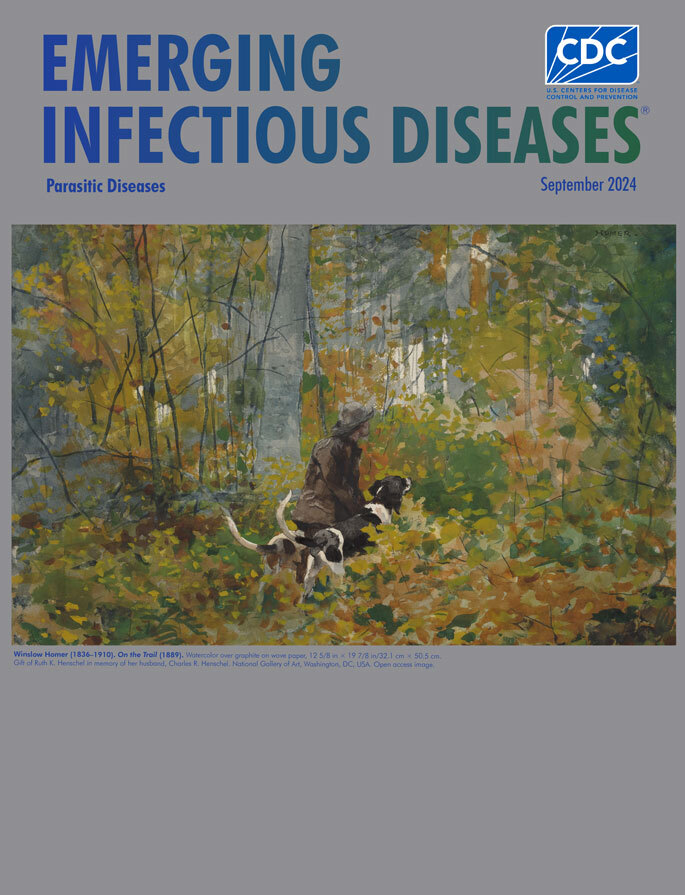
**Winslow Homer (1836–1910). *On the Trail* (1889).** Watercolor over graphite on wove paper, 12 5/8 in × 19 7/8 in/32.1 cm × 50.5 cm. Gift of Ruth K. Henschel in memory of her husband, Charles R. Henschel. National Gallery of Art, Washington, DC, USA. Open access image.

“Winslow Homer is regarded by many as the greatest American painter of the nineteenth century,” noted art historian and curator H. Barbara Weinberg. Homer’s skill at mastering watercolor and oil painting is what led to his being considered among America’s foremost painters.

Homer was born in Boston, Massachusetts, USA, and grew up in nearby Cambridge. He received no formal artistic education, although his mother instructed him in watercolor. He initially apprenticed as a commercial printmaker in Boston and continued in that trade after moving to New York, New York, USA, in 1859. Commercial printmaking was a profession that Homer grew to dislike. In New York, he first freelanced as an illustrator for magazines, including *Harper’s Weekly*, briefly took drawing classes at the National Academy of Design, and began to focus on painting.

During the US Civil War, when Homer was 25 years old, *Harper’s Weekly* dispatched him as an artist-correspondent embedded with the Union army, and he gained widespread recognition for his work. “Homer gave people views of the war that most would never see,” according to Keely Orgeman, the Seymour H. Knox, Jr., Associate Curator of Modern and Contemporary Art at the Yale University Art Gallery, quoted in an article in *Yale News.*

Homer spent 1867 abroad in France, starting to paint in watercolor in 1873, and in 1875 he ended his career as a magazine illustrator to focus on painting. In 1881, Homer moved to the seaside village of Cullercoats, England, an experience that preceded his permanent move in 1883 to the fishing village of Prouts Neck, Maine, USA. On that coastal peninsula, Homer’s studio, converted from a stable, provided him both the solitude and scenery he craved.

In a 1923 collection of Homer’s work, painter and art writer Nathaniel Pousette-Dart asserted, “Certainly no artist in whose work the influence of contemporary painters is less apparent ever lived. He shunned exhibits and rarely contributed his own pictures to them…. He lived during the greater part of his life like a hermit.” Although Homer lived in Prouts Neck for the rest of his life, he frequently wintered in Florida and Bermuda and often visited Boston and the Adirondack region of upstate New York. Among his favorite subjects were fishermen and hunters.

This month’s cover image, *On the Trail,* is among Homer’s New York paintings. The dappled light and gossamer layers of leaves, rendered as splotches of yellow, orange, and green, suggest early autumn, show the artist’s mastery at rendering scenes from the natural world. An attentive young hunter peers into the thick forest, clutching his eager dogs, anticipating an unsuspecting deer. The quiet intensity Homer conveys diverges from more dramatic scenes in many of his other hunting-themed paintings. The National Gallery of Art notes, “These works celebrate the pleasures and beauty of life in the Adirondacks but also confront the more brutal realities of hunting. In one series, Homer depicted a practice called hounding, in which dogs were used to drive deer into a lake.”

In *On the Trail*, the fate of the unseen deer remains unsettled; perhaps it escaped in the underbrush. Regardless, the young hunter would likely have given little thought to health threats in such sylvan settings, particularly from ticks. Health threats from tickborne pathogens were not as widespread or recognized then as they are now. Homer’s hunter, dogs, and unseen deer would have been less likely than modern hunters and hikers to encounter ticks that transmit diseases. Epidemiologist Katharine Walters and colleagues, in their article “Genomic Insights into the Ancient Spread of Lyme Disease across North America Deforestation” note that urbanization, climate change, and increased deer populations have altered the ecology and landscape, enabling ticks to flourish and spread.

Ticks are vectors for numerous bacterial diseases, including anaplasmosis, bartonellosis, ehrlichiosis, Lyme disease, and spotted fever rickettsioses. They are also vectors for viral diseases including Bourbon virus disease, Colorado tick fever, Crimean-Congo hemorrhagic fever, Heartland virus disease, and Powassan encephalitis. Tick bites may also transmit parasitic diseases such as babesiosis, a disease caused by microscopic parasites that infect red blood cells. Most cases of babesiosis are caused by *Babesia microti*, transmitted in North America by bites from *Ixodes scapularis* ticks. The ticks that cause that parasitic disease, now endemic to many places Homer lived and visited in the northeastern United States, have expanded their range from Virginia to Maine and the upper midwestern states. As reported in this issue of *Emerging Infectious Diseases*, including in a new report from Hungary and another in the Netherlands, nonimported human babesiosis is increasing its range in Europe.

Articles about *Mansonella* *ozzardi*, a filarial worm transmitted by biting midges and black flies, in raccoons in urban areas of Costa Rica and emergence of *Thelazia callipaeda*, an ocular nematode of carnivores and humans, in black bears in Pennsylvania, USA, document the work of researchers on the trail of potential spillover pathogens. Through his art, Homer afforded views of the Civil War, the ocean, and the woods that most people would never experience. Through their epidemiologic sleuthing, researchers tracking emerging vectorborne health threats provide public health authorities with views most would never have and help respond to those threats.
